# *Bordetella bronchiseptica*: a rare cause of meningitis

**DOI:** 10.1186/s12879-020-05668-2

**Published:** 2020-12-03

**Authors:** Christopher Radcliffe, Audun Lier, Natnael Doilicho, Sunil Parikh, Firas Kaddouh

**Affiliations:** 1 Yale School of Medicine, 15th York Street, LLCI 10th floor, P.O. Box 208018, New Haven, CT 06520 USA; 2 Department of Internal Medicine, Section of Infectious Diseases, Yale New Haven Hospital, New Haven, CT USA; 3 Department of Neurology, Division of Neurocritical Care and Emergency Neurology, Yale New Haven Hospital, New Haven, CT USA

**Keywords:** *Bordetella bronchiseptica*, Infliximab, Meningitis, Emerging infections

## Abstract

**Background:**

*Bordetella bronchiseptica* is a gram-negative, obligate aerobic coccobacillus known to cause disease in domesticated animals and pets. In humans, *B. bronchiseptica* commonly leads to respiratory infections like pneumonia or bronchitis, and animal contact usually precedes the onset of symptoms.

**Case presentation:**

We report a case of post-traumatic *B. bronchiseptica* meningitis without recent surgery in the setting of immunosuppression with a monoclonal antibody. Our case concerns a 77-year-old male with ulcerative colitis on infliximab who sustained a mechanical fall and developed a traumatic cerebrospinal fluid leak complicated by meningitis. He received meropenem then ceftazidime during his hospital course, and temporary neurosurgical drain placement was required. His clinical condition improved, and he was discharged at his baseline neurological status.

**Conclusions:**

*B. bronchiseptica* is an unusual cause of meningitis that may warrant consideration in immunocompromised hosts with known or suspected animal exposures. To better characterize this rare cause of meningitis, we performed a systematic literature review and summarized all previously reported cases.

## Background

First isolated in 1911 as the causative agent behind canine distemper [1], *Bordetella bronchiseptica* is a gram-negative, obligate aerobe known to cause disease in dogs, pigs, and several other animals [2]. In humans, animal contact is often the source of transmission, and respiratory infections like pneumonia or bronchitis are common clinical syndromes [2]. *B. bronchiseptica* infections often occur in people with immunocompromising conditions [3–9], and the majority of patients in a recent series had comorbidities which increased their susceptibility to infection [10].

Unlike respiratory infections, *B. bronchiseptica* meningitis is exceedingly rare [11–13]. We report a case of a 77-year-old male with ulcerative colitis on infliximab who sustained a fall and developed a traumatic cerebrospinal fluid (CSF) leak complicated by *B. bronchiseptica* meningitis. To our knowledge, this represents the first reported case of post-traumatic *B. bronchiseptica* meningitis in a patient without recent central nervous system instrumentation. We performed a systematic literature review and summarized all prior reports in order to better characterize this potentially emerging cause of meningitis.

## Case presentation

A 77-year-old male with a past medical history of hypertension, osteoarthritis, and ulcerative colitis on infliximab presented to an outside hospital after an unwitnessed mechanical fall. There was no noted preceding prodrome. He was retired and volunteered frequently at animal shelters with extensive animal contact. On presentation, he endorsed a headache as well as posterior neck pain and was found to be hypertensive (systolic blood pressure 208 mmHg) with leukocytosis (21,100/μL; ref. range 3500-10,000/μL). A head-and-neck computed tomography (CT) scan revealed small subdural hematomas, mild traumatic subarachnoid hemorrhage with small intraventricular component, and a nondisplaced fracture of the sphenoid sinus.

He was admitted to the intensive care unit (ICU) for management of hypertensive emergency and was treated empirically with a 7-day course of intravenous ampicillin-sulbactam for presumed aspiration pneumonia. Sputum cultures were not obtained. On hospital day 8, he developed serosanguinous nasal drainage, confirmed to be CSF rhinorrhea by beta-2-transferrin testing, which subsequently resolved without intervention. While on the floor, he was ambulating and functioning at baseline per family members, but developed mild dizziness and new headache on hospital day 10. The following morning, he exhibited altered mental status with a temperature of 39.2 °C and blood pressure of 230/110 mmHg. His markedly elevated blood pressure was attributed to agitation, suspected seizure, and poorly-controlled hypertension at baseline. Laboratory studies revealed new leukocytosis (16,000/μL). Blood cultures were drawn and returned negative. A repeat CT scan was stable without findings suggestive of herniation or significant cerebral edema, and CSF cultures were collected via lumbar puncture. Intravenous meropenem was empirically started, and he was readmitted to the ICU for neurological monitoring and blood pressure control. Blood cultures were again drawn on hospital day 12 and returned negative.

*B. bronchiseptica* eventually grew on CSF culture, and meropenem was continued. Table 1 lists the isolate’s susceptibility profile. On hospital day 17, he had worsening encephalopathy, a repeat CSF analysis showed resolving pleocytosis, and a repeat CT scan showed stable communicating hydrocephalus. He was transferred to Yale New Haven Hospital the following day. On hospital day 19, temporary CSF diversion was achieved by a lumbar drain due to concern for communicating hydrocephalus. CSF cultures were collected at the time of lumbar drain placement and returned negative. Three subsequent CSF cultures were also negative. On hospital day 20, sensitivity data for *B. bronchiseptica* cultures were received from the outside hospital, and antibiotic therapy was deescalated to intravenous ceftazidime. Three days after its placement, the lumbar drain was removed and replaced with an external ventricular drain since the patient’s neurological exam failed to improve. A CT scan demonstrated persistent ventriculomegaly, and obstructive hydrocephalus was suspected. His mental status dramatically improved over the following days, and the external ventricular drain was removed 7 days after placement. Notably, ventriculomegaly persisted on a repeat CT scan. Guided by CSF profile and culture data, ceftazidime was discontinued at that time. In total, he received 10 days of meropenem and an additional 9 days of ceftazidime. He was at his baseline neurologically when discharged to a rehabilitation facility after a 32-day hospital stay.

**Table 1:** Sensitivity data for *B. bronchiseptica* isolate

Susceptible	Intermediate	Resistant
ceftazidime	cefepime	aztreonam
gentamicin	ceftriaxone	
imipenem		
piperacillin-tazobactam		
tobramycin		
trimethoprim/sulfamethoxazole		

Legend: The isolate’s susceptibility profile was determined using a DxM MicroScan WalkAway (Beckman Coulter) instrument. Bold text represents column titles

## Discussion and conclusions

*B. bronchiseptica* commonly causes respiratory infections when transmitted to humans [2]. To our knowledge, this is the first case of non-surgical *B. bronchiseptica* meningitis complicating a traumatic CSF leak. Our patient was successfully treated with a 19-day course of antibiotics and temporary CSF diversion. It is presumed that his exposure to *B. bronchiseptica* occurred during his volunteer activities at local animal shelters, but it is unknown whether he was colonized with *B. bronchiseptica*, as previous studies support *B. bronchiseptica* colonization of the human respiratory tract [10, 14]. Both his advanced age and infliximab therapy may have contributed to his immunocompromised status and increased susceptibility to infection. Ultimately, he survived the hospital stay with significant recovery and returned to his baseline neurological status.

To search PubMed and Ovid databases for *B. bronchiseptica* meningitis cases, we used the following search operators: (“Bordetella bronchiseptica” OR “Haemophilus bronchiseptica” OR “Brucella bronchiseptica” OR “Bacillus suisepticus” OR “*Alcaligenes bronchicanis*” OR “Alcaligenes bronchisepticus”) AND (meningitis OR encephalitis OR cerebritis OR “cerebral abscess”). Table 2 summarizes all reported cases and the present case. Two pediatric cases and one adult case have been reported [11–13]. The first report concerned a 9-year-old male who underwent an open reduction for orbital fractures after being kicked in the head by a horse [11], another was a case of a 17-year-old female involving an acoustic neuroma resection 6 weeks before presentation [12], and the only previously reported adult case concerned a 49-year-old male who had a glioblastoma resected 6 weeks before presentation [13]. Treatment was successful for both pediatric cases [11, 12], and the 49-year-old male died after transitioning to palliative care [13].

**Table 2:** Summary of reported *B. bronchiseptica* meningitis cases

Age/Sex	Animal Exposure	Recent Central Nervous System Instrumentation	Recent Trauma	Initial Cerebrospinal Fluid Profile	Outcome	Reference
9-year-old/male	household pets	open reduction procedure for orbital fractures	head trauma from horse kick	white blood cell count, 2034/mm^3^; no red blood cells; glucose, 55 mg/dL; protein, 40 mg/dL	treatment success	[11]
17-year-old/female	household pets	acoustic neuroma resection	none	white blood cell count, 20/mm^3^; glucose, 49 mg/dL; protein, 25 mg/dL; Gram-stain negative	treatment success	[12]
49-year-old/male	household pets	glioblastoma resection	none	white blood cell count, 28; red blood cell count, 8; glucose, 59 mg/dL; protein, 26 mg/dL; Gram-stain negative	transitioned to palliative care due to underlying glioblastoma and died	[13]
77-year-old/male	animal shelters	none	mechanical fall	white blood cell count, 1700; red blood cell count, 103; glucose, 95 mg/dL (serum glucose 178 mg/dL); protein, 95 mg/dL; Gram-stain negative	treatment success	our case

Legend: The table summarizes all reported cases identified by searching PubMed and Ovid databases. Bold text represents column titles

Our case is notable for being the first reported case of *B. bronchiseptica* meningitis without surgical instrumentation preceding the initial presentation; however, the infection in the 9-year-old male may have been subclinical after sustaining head trauma and only manifested after surgery [11]. All previously reported cases had documented animal exposures [11–13], and our case is consistent with these reports. Interestingly, the household pets and horse involved in the 9-year-old male’s case tested negative for *B. bronchiseptica*, and the true source of his infection was unknown [11]. Although rare, *B. bronchiseptica* meningitis has now been reported in several patients with compromised meningeal barriers and immunocompromising comorbidities, all with suspected animal exposures [12, 13]. Furthermore, the increasing use of immunosuppressive agents for the treatment of autoimmune, oncological, or cardiovascular diseases merits increased vigilance for uncommon causes of meningitis or systemic infection [15–17].

## Data Availability

Materials in the form of electronic medical records are available but unable to be released due to the Health Insurance Portability and Accountability Act. All relevant, deidentified data has been presented in this case report and further inquiry can be addressed to the corresponding author.

## Abbreviations

CSF: Cerebrospinal fluid
CT: Computed tomography
ICU: Intensive care unit

## References

[1] Ferry NS (1911). Etiology of canine distemper. J Infect Dis.

[2] Woolfrey BF, Moody JA (1991). Human infections associated with Bordetella bronchiseptica. Clin Microbiol Rev.

[3] Dworkin MS, Sullivan PS, Buskin SE, Harrington RD, Olliffe J, MacArthur RD (1999). Bordetella bronchiseptica infection in human immunodeficiency virus-infected patients. Clin Infect Dis.

[4] Woods P, Ordemann K, Stanecki C, Brown J, Uzodi A (2020). Bordetella bronchiseptica pneumonia in an adolescent: case report and review of the pediatric literature. Clin Pediatr (Phila).

[5] Powers HR, Shah K. Bordetella bronchiseptica bloodstream infection in a renal transplant patient. Transpl Infect Dis. 2017;19(6). 10.1111/tid.12774 Epub 2017 Oct 25. 10.1111/tid.12774 28865149

[6] Mazumder SA, Cleveland KO (2010). Bordetella bronchiseptica bacteremia in a patient with AIDS. South Med J.

[7] Redelman-Sidi G, Grommes C, Papanicolaou G (2011). Kitten-transmitted Bordetella bronchiseptica infection in a patient receiving temozolomide for glioblastoma. J Neuro-Oncol.

[8] Gisel JJ, Brumble LM, Johnson MM (2010). Bordetella bronchiseptica pneumonia in a kidney-pancreas transplant patient after exposure to recently vaccinated dogs. Transpl Infect Dis.

[9] Bauwens JE, Spach DH, Schacker TW, Mustafa MM, Bowden RA (1992). Bordetella bronchiseptica pneumonia and bacteremia following bone marrow transplantation. J Clin Microbiol.

[10] Garcia-de-la-Fuente C, Guzman L, Cano ME, Aguero J, Sanjuan C, Rodriguez C (2015). Microbiological and clinical aspects of respiratory infections associated with Bordetella bronchiseptica. Diagn Microbiol Infect Dis.

[11] Chang KC, Zakhein RM, Cho CT, Montgomery JC (1975). Letter: posttraumatic purulent meningitis due to Bordetella bronchiseptica. J Pediatr.

[12] Belen O, Campos JM, Cogen PH, Jantausch BA (2003). Postsurgical meningitis caused by Bordetella bronchiseptica. Pediatr Infect Dis J.

[13] Groner M, Rodriguez A, Doblecki-Lewis S (2016). Bordetella bronchiseptica post-surgical meningitis in an adult. Infect Dis Clin Pract.

[14] Wernli D, Emonet S, Schrenzel J, Harbarth S (2011). Evaluation of eight cases of confirmed Bordetella bronchiseptica infection and colonization over a 15-year period. Clin Microbiol Infect.

[15] Cholapranee A, Hazlewood GS, Kaplan GG, Peyrin-Biroulet L, Ananthakrishnan AN (2017). Systematic review with meta-analysis: comparative efficacy of biologics for induction and maintenance of mucosal healing in Crohn's disease and ulcerative colitis controlled trials. Aliment Pharmacol Ther.

[16] Monaco C, Nanchahal J, Taylor P, Feldmann M (2015). Anti-TNF therapy: past, present and future. Int Immunol.

[17] Salvana EM, Salata RA (2009). Infectious complications associated with monoclonal antibodies and related small molecules. Clin Microbiol Rev.

